# Plasmid-Borne AFM Alleles in Pseudomonas aeruginosa Clinical Isolates from China

**DOI:** 10.1128/spectrum.02035-22

**Published:** 2022-08-24

**Authors:** Minhua Chen, Heng Cai, Yue Li, Nanfei Wang, Piaopiao Zhang, Xiaoting Hua, Yunsong Yu, Renhua Sun

**Affiliations:** a Emergency and Critical Care Center, Intensive Care Unit, Zhejiang Provincial People's Hospital (Affiliated People's Hospital, Hangzhou Medical College), Hangzhou, Zhejiang, People’s Republic of China; b Department of Infectious Diseases, Sir Run Shaw Hospital, Zhejiang University School of Medicine, Hangzhou, People’s Republic of China; c Key Laboratory of Microbial Technology and Bioinformatics of Zhejiang Province, Hangzhou, People’s Republic of China; d Regional Medical Center for National Institute of Respiratory Diseases, Sir Run Shaw Hospital, School of Medicine, Zhejiang University, Hangzhou, People’s Republic of China; Brown University

**Keywords:** *Pseudomonas aeruginosa*, metallo-β-lactamase, AFM-2, AFM-4, plasmid

## Abstract

Carbapenem-resistant Pseudomonas aeruginosa (CRPA) is a pathogen of global concern due to the fact that therapeutic drugs are limited. Metallo-β-lactamase (MBL)-producing P. aeruginosa has become a critical part of CRPA. Alcaligenes faecalis metallo-β-lactamase (AFM) is a newly identified subclass B1b MBL. In this study, 487 P. aeruginosa strains isolated from patients and the environment in an intensive care unit were screened for AFM alleles. Five AFM-producing strains were identified, including four AFM-2-producing strains (ST262) and one AFM-4-producing strain (ST671). AFM-2-producing strains were isolated from rectal and throat swabs, and AFM-4-producing strains were isolated from the water sink. The *bla*_AFM-2_ carrying plasmids belonged to the IncP-2 type, while the *bla*_AFM-4_ carrying plasmid pAR19438 was a pSTY-like megaplasmid. Plasmid pAR19438 was acquired *bla*_AFM-4_ by the integration of the Tn*1403*-like transposon. All *bla*_AFM_ genes were embedded in an IS*CR29*-*bla*_AFM_ unit core module flanked by class 1 integrons. The core module of *bla*_AFM-2_ was IS*CR29*-Δ*groL*-*bla*_AFM-2_-*ble*_MBL_-Δ*trpF*-ΔIS*CR*, while the core module of *bla*_AFM-4_ was IS*CR29*-Δ*groL*-*bla*_AFM-2_-*ble*_MBL_-Δ*trpF*-IS*CR*-*msrB*-*msrA*-*yfcG*-*corA*-ΔIS*CR*. The flanking sequences of IS*CR29*-*bla*_AFM_ units also differed. The expression of AFM-2 and AFM-4 in DH5α and PAO1 illustrated the same effect for the evaluation of the MICs of β-lactams, except for aztreonam. Identification of AFM-4 underscores that the quick spread and emerging development of mutants of MBLs require continuous surveillance in P. aeruginosa.

**IMPORTANCE** Acquiring metallo-β-lactamase genes is one of the important carbapenem resistance mechanisms of P. aeruginosa. Alcaligenes faecalis metallo-β-lactamase is a newly identified metallo-β-lactamase, the prevalence and genetic context of which need to be explored. In this study, we identified AFM-producing P. aeruginosa strains among clinical isolates and found a new mutant of AFM, AFM-4. The *bla*_AFM-4_ carrying plasmid pAR19438 was a pSTY-like megaplasmid, unlike the plasmids encoding other *bla*_AFM_ alleles. The genetic context of *bla*_AFM-4_ was also different. However, AFM-2 and AFM-4 had the same impacts on antibiotic susceptibility. The presence and transmission of AFM alleles in P. aeruginosa pose a challenge to clinical practice.

## INTRODUCTION

Carbapenem-resistant Pseudomonas aeruginosa (CRPA) is a threat to global public health and is listed in the critical priority for the requirement of new antibiotics (1). P. aeruginosa can develop resistance to carbapenems in multiple ways, and acquiring carbapenemase genes is one of the most important carbapenem resistance mechanisms (2). Worldwide, Ambler B class metallo-β-lactamase (MBL) genes are the most prevalent type of carbapenemase genes in P. aeruginosa (3, 4). MBLs can hydrolyze most β-lactams except monobactams but cannot be inhibited by novel β-lactamase inhibitors such as avibactam, vaborbactam, relebactam, and nacubactam (5). Thus, treatments for MBL-producing P. aeruginosa infections are very limited. Numerous types of MBLs have been reported. In P. aeruginosa, Verona integron–encoded metallo-β-lactamases (VIMs), imipenemases (IMPs), and New Delhi metallo-β-lactamases (NDMs) are the most frequently identified types (6).

Alcaligenes faecalis metallo-β-lactamase-1 (AFM-1), a novel subclass B1b MBL, was first identified in Alcaligenes faecalis in China in 2018 (GenBank accession: MK143105). Subsequently, AFM-1 was identified in Comamonas testosteroni, Comamonas aquatica, Bordetella trematum, P. aeruginosa, P. putida, and Stenotrophomonas maltophilia (GenBank accessions: MT011984, MT180074, CP049957, CP061377, MN699650, and CP049956, respectively), and AFM-2 and AFM-3 were identified in Pseudomonas aeruginosa (7, 8). To date, there are few reports about this newly identified MBL-producing organism.

In this study, we screened AFM alleles from 487 P. aeruginosa strains isolated from an intensive care unit (ICU) in Zhejiang Provincial People's Hospital in Zhejiang, China. Five AFM-producing P. aeruginosa strains were identified, including one strain carrying a novel AFM allele AFM-4. The genetic characteristics of these strains were analyzed and the effect of AFM-4 on antibiotic resistance was evaluated.

## RESULTS

### Identification of AFM-carrying strains.

We collected 487 P. aeruginosa isolates from environmental and patient samples in the ICU of Zhejiang Provincial People's Hospital, between April and July 2021. We screened these isolates for AFM alleles and consequently found five AFM-carrying P. aeruginosa strains, named AR19438, AR19640, AR19726, AR19727, and AR23664. AR19438 was isolated from the surface of a water sink in the ward. The other specimens were isolated from the same patient within 45 days: AR19640 and AR19726 were isolated from rectal swabs, and AR19727 and AR23664 were isolated from throat swabs. Genome sequencing revealed that AR19438 belonged to sequence type (ST) 671 and that the other four strains belonged to ST262. Using PCR and sequencing, we identified AR19438 as a novel AFM allele, named AFM-4 (GenBank accession: OM049002), while the other strains had AFM-2. In the amino acid sequence of AFM-2, the valine 15 residue is replaced with alanine (GenBank accession: AYV97588), while in the amino acid sequence of AFM-4, the proline 13 residue is replaced with leucine (Fig. S1).

### Results of antimicrobial susceptibility testing.

For antimicrobial susceptibility testing, we included another three relative strains without AFM for comparison (Table 1). AR19437 belonging to ST671 was isolated from the scupper of the water sink, and on the same day, AR19438 was isolated. AR19538 and AR23663 were isolated from rectal swabs of the same patient from whom the other ST262 strains were isolated.

**TABLE 1:** Information on strains collected from the ICU

Strain	Isolation date (yr-mo-day)	Bed unit	Sample type	AFM alleles	Sequence type
AR19437	2021-05-19	23	Water sink scupper		671
AR19438	2021-05-19	23	Water sink surface	AFM-4	671
AR19583	2021-06-16	23	Rectal swab		262
AR19640	2021-06-30	23	Rectal swab	AFM-2	262
AR19726	2021-07-14	23	Rectal swab	AFM-2	262
AR19727	2021-07-14	23	Throat swab	AFM-2	262
AR23663	2021-07-28	23	Rectal swab		262
AR23664	2021-07-28	23	Throat swab	AFM-2	262

Both AR19437 and AR19438 are susceptible to aztreonam, amikacin, gentamicin, and colistin. AR19438 is resistant to meropenem, imipenem, cefepime, piperacillin, ceftazidime, piperacillin-tazobactam, ceftazidime-avibactam, and ciprofloxacin, while AR19437 showed various susceptibility to these antibiotics. All AFM-2-carrying strains were resistant to most tested antibiotics except for aztreonam and colistin, and the latter was active against all isolates. For aztreonam, AR23663 is susceptible, AR19640 and AR19726 show intermediate susceptibility, and AR19727 is resistant. The ST262 strains without AFM-2 are susceptible to carbapenems, aminoglycosides, ciprofloxacin, and colistin but resistant to the β-lactams and β-lactam combination agents listed (Table 2).

**TABLE 2:** Antimicrobial susceptibility testing results of the clinical isolated strains^*a*^

	MICs of antibiotics (mg/L)											
Strains	MEM	IPM	FEP	PIP	CAZ	PTZ	CZA	AZT	CIP	AMK	GEN	COL
AR19437	0.125	4	4	16	16	8/4	8/4	4	0.125	4	2	0.5
AR19438	32	128	>256	256	>256	256/4	>256/4	4	8	4	2	0.25
AR19583	1	2	64	256	256	256/4	64/4	64	0.125	4	2	0.25
AR19640	64	>128	>256	256	>256	256/4	>256/4	16	8	>64	>64	0.25
AR19726	128	>128	>256	256	>256	256/4	>256/4	16	8	>64	>64	0.25
AR19727	128	128	>256	256	>256	256/4	>256/4	64	8	>64	>64	0.25
AR23663	1	1	32	256	256	>256/4	64/4	32	0.125	8	2	0.25
AR23664	32	64	>256	128	>256	128/4	>256/4	8	8	>64	>64	0.25

*^a^*MEM, meropenem; IPM, imipenem; FEP, cefepime; PIP, piperacillin; CAZ, ceftazidime; PTZ, piperacillin-tazobactam; CZA, ceftazidime-avibactam; AZT, aztreonam; CIP, ciprofloxacin; AMK, amikacin; GEN, gentamicin; COL, colistin.

### The characteristics of AFM-carrying plasmids.

**FIG 1:**
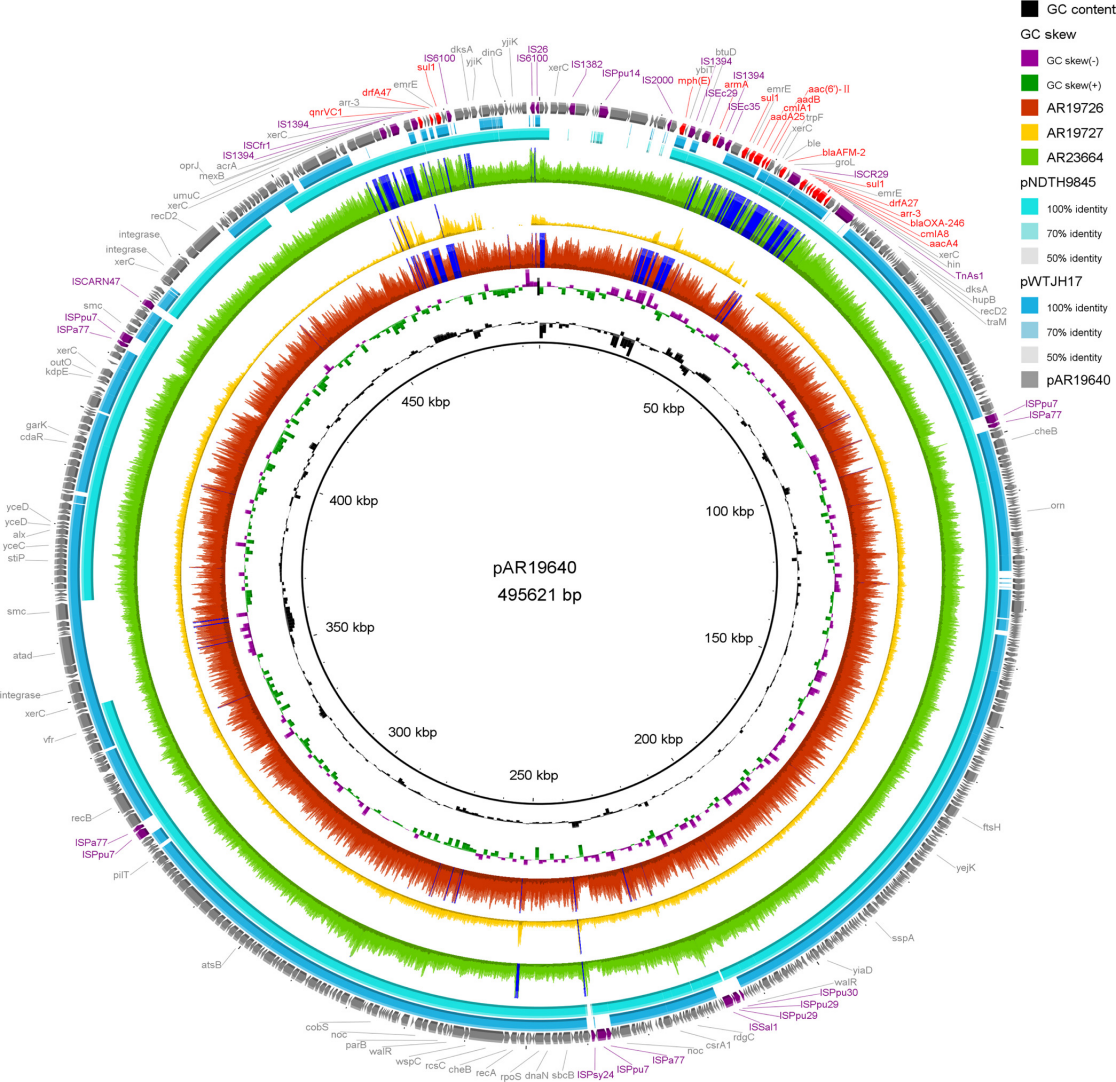
Comparison of pAR19640 and other plasmids. The rings of AR19726, AR19727 and AR23664 were the alignment of sequence reads against pAR19640. pNDTH9845 and pWTJH17 contain *bla*_AFM-2_ and *bla*_AFM-3_, respectively.

Whole-genome sequencing revealed that *bla*_AFM-2_ and *bla*_AFM-4_ were embedded in two distinct plasmids. As indicated by alignment, all of the AFM-2 carrying strains contained plasmids that were quite similar to the sole plasmid pAR19640 in AR19640 (Fig. 1). Plasmid pAR19640 shares 90% and 80% query coverage with pNDTH9845 (GenBank accession: CP073081) and pWTJH17 (GenBank accession: CP073083), respectively. The backbones of these homologous plasmids were similar to those of the IncP-2 plasmid pOZ176, indicating that they belonged to the same megaplasmid family (9). The plasmid pAR19640 is 49562 bp long with an average GC content of 56%. There are numerous resistance genes embedded in pAR19640, which can roughly be divided into two gene clusters. One resistance gene cluster located from approximately 25 kbp to 55 kbp contains most of the resistance genes, including *bla*_AFM-2_. The other resistance gene cluster contains only eight predicted open reading frames flanked by IS*1394* and IS*6100* (approximately 480 kbp). The differences among pAR19640, pNDTH9845 and pWTJH17 are linked to the presence of insertion sequences (ISs) or genes encoding integrase, indicating that these plasmids evolved through multiple insertions and recombinations.

**FIG 2:**
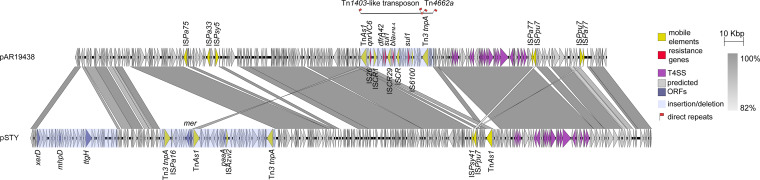
Comparison of pAR19438 and pSTY. The arrow direction indicates the transcription direction of each ORF. The dark yellow arrows indicate mobile elements. The red arrows indicate resistance genes. The gray and blue arrows indicate predicted ORFs. The blue shades indicate long-segment insertions/deletions. The black line indicates transposons. The red block on the line indicates the 163 bp gap between the transposons. The red flags indicate direct repeats.

**FIG 3:**
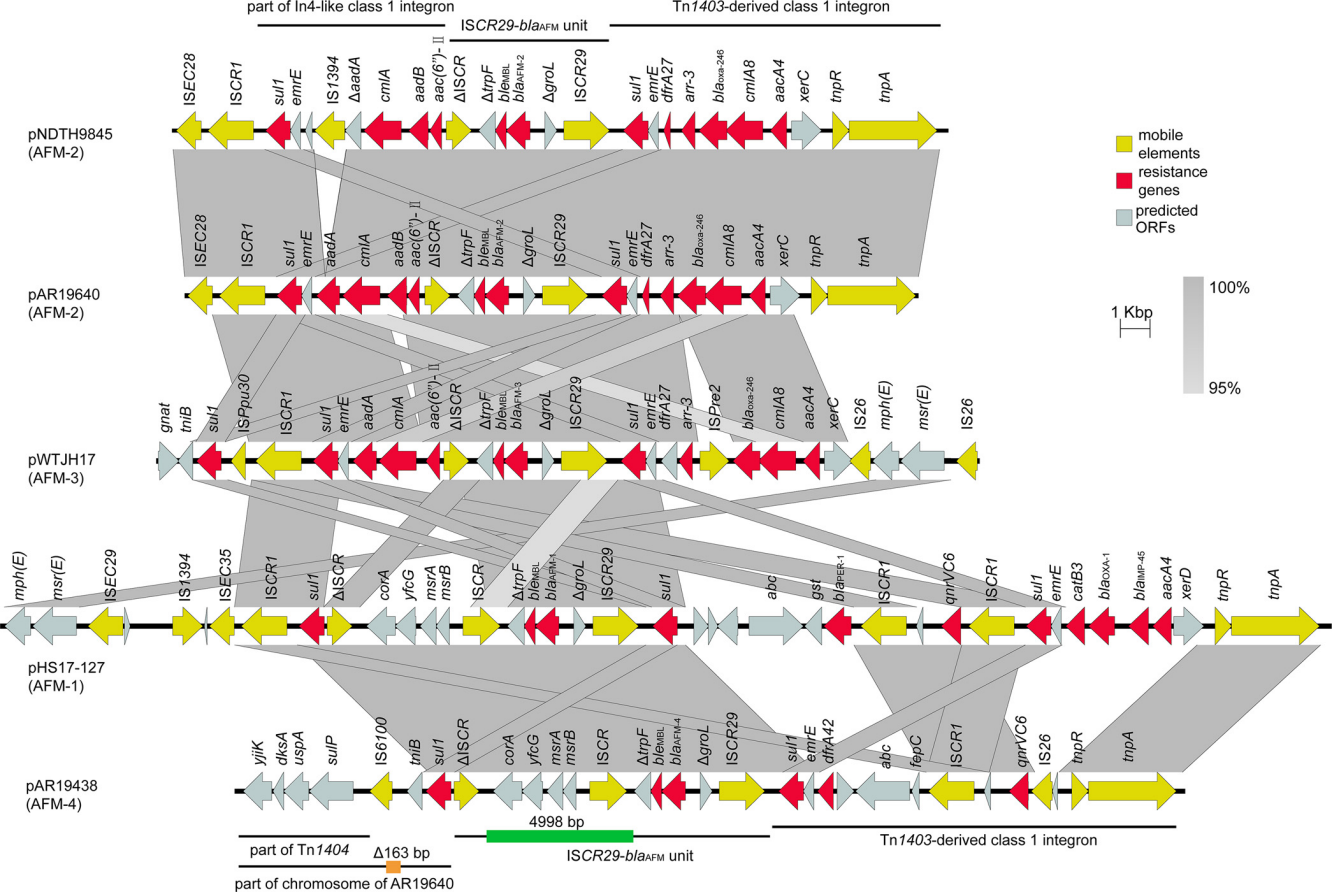
The genetic contexts of *bla*_AFM_ genes in P. aeruginosa. The arrow direction indicates the transcription direction of each ORF. The dark yellow arrows indicate mobile elements. The red arrows indicate resistance genes. The light blue arrows indicate predicted ORFs. The integrons, transposon and IS*CR29*-*bla*_AFM_ unit are marked by black lines. The black line indicates that the marked part of pAR19438 is similar to part of the chromosome of AR19640, and the orange block on the line indicates a 163 bp deletion. The green block on the line indicates a 4998 bp insertion in the IS*CR29*-*bla*_AFM-4_ unit compared to the IS*CR29*-*bla*_AFM-2_ unit.

AFM-4 carrying plasmid pAR19438 is 275,369 bp long with an average GC content of 56%. Among several homologous plasmids found in NCBI, pAR19438 is most similar to pSTY (GenBank accession: CP003962), which is a megaplasmid conserved by Pseudomonas taiwanensis strain VLB120 that renders solvent tolerance (10, 11). pAR19438 shares 75% query coverage and 99.98% identity with pSTY by BLAST. The common parts of the two plasmids encode the type IV secretion system (T4SS) and plasmid maintenance proteins. However, there were three long-segment insertions/deletions between pAR19438 and pSTY (Fig. 2). The first deletion in pAR19438 begins with *xerD* encoding phage integrase site specific recombinase and ends approximately 41 kbp downstream. This part of the sequence includes genes encoding enzymes such as 2-keto-4-pentenoate hydratase (*mphD*) and toluene efflux pump gene cluster *ttgGHI*. The second deletion in pAR19438 includes mercury-tolerant operon (*mer*) and phenylacetic acid degradative pathway genes (*paa* genes) and there are two Tn3-family transposase genes (*tnpA*) close to both ends. These two gene segments in pSTY provide VLB120 with solvent tolerance. The last difference is the long-segment insertion in pAR19438 compared to pSTY, which consists of abundant mobile genetic elements and antimicrobial resistance genes, including *bla*_AFM-4_. This long-segment insertion includes two transposons, Tn*1403*-like transposon and Tn*4662a*, with a 163 bp gap between them (Fig. 2). Tn*1403*-like transposon is 31,682 bp in length flanked by 7 bp direct repeats (5′-ctctctg-3′) including *bla*_AFM-4_ and its surrounding sequences, which are concretely shown in Fig. 3. Tn*4662a* is 5,635 bp in length flanked by 7 bp direct repeats (5′-aacatgg-3′) including genes encoding DNA invertase, RelE/ParE family toxin, helix-turn-helix protein, etc.

We used oriTfinder to identify four components of a conjugative apparatus in pAR19438 and pAR19640, and only identified the T4SS in pAR19438 (Fig. 2). We conducted conjunction assays to determine the transferability of pAR19640 and pAR19438. The experiments were performed three times but all failed, suggesting that these plasmids are not conjugative.

### Genetic contexts of *bla*_AFM_ alleles in P. aeruginosa.

All AFM alleles have been reported in P. aeruginosa strains thus far (7, 8). We chose pNDTH9845, pWTJH17, pHS17-127 (GenBank accession: CP061377), and two AFM-carrying plasmids in this study to compare the genetic contexts of AFM alleles in P. aeruginosa (Fig. 3).

The genetic contexts of *bla*_AFM-2_ and *bla*_AFM-3_ are both an IS*CR29*-*bla*_AFM_ unit flanked by two complex class 1 integrons indicates. The IS*CR29*-*bla*_AFM_ units of *bla*_AFM-2_ and *bla*_AFM-3_ sequentially consist of IS*CR29*, Δ*groL*, *bla*_AFM_, *ble*_MBL_, Δ*trpF*, and ΔIS*CR*. In pAR19640, the downstream region of the IS*CR29*-*bla*_AFM_ unit is part of an In4-like class 1 integron, IS*CR1* and IS*EC28*. The In4-like class 1 integron contains resistance genes, including *sul1*, *aadA*, *cmlA*, *aadB*, and *aac(6”)-II*. In pNDTH9845, *aadA* was disrupted by IS*1394*. In pWTJH17, *aadB* was missing and the region downstream of IS*CR1* was further replaced by IS*Ppu30*, *sul1*, *tniB,* and *gnat*. The upstream part of the IS*CR29*-*bla*_AFM_ unit in pAR19640 is a Tn*1403*-derived class 1 integron, which is identical to pNDTH9845. This integron contains resistance genes, including *sul1*, *dfrA27*, *arr-3*, *bla*_OXA-246_, *cmlA8,* and *aacA4*. The upstream part in pWTJH17 is different from that of pAR19640 in that an IS*Pre2* has been inserted in the intergenic region between *arr-3* and *bla*_OXA-246_, and *tnpA-tnpR* has been replaced by IS*26*-induced insertion carrying *msr(E)-mph(E)* (macrolide resistance genes).

The genetic context of *bla*_AFM-4_ is significantly different from those of *bla*_AFM-2_ and *bla*_AFM-3_. First, the core module IS*CR29*-*bla*_AFM-4_ unit is identical to the unit in pHS17-127, in which there is a 4998 bp insertion of putative IS*CR* with the cassette array *msrB*-*msrA*-*yfcG*-*corA*. Second, the 7091 bp-long sequence downstream of the IS*CR29*-*bla*_AFM_ unit is coincidentally identical to part of pAR19640 (ranging from 478504 bp to 485435 bp), except for the 163 bp-long deletion at 61 bp upstream of IS*6100*. This region includes the cassette array *sulP*-*uspA*-*dksA*-*yiiK*, which can be found in Tn*1404* (GenBank accession: AH008062) and Tn*6609* (GenBank accession: MZ361367). Finally, the upstream region of the IS*CR29*-*bla*_AFM_ unit is a different Tn*1403*-derived class 1 integron from pAR19640, since they share the same transposase and 3′ conserved sequences but contain different passenger segments. The passenger segments include *dfrA42* (trimethoprim resistance gene), *abc* (gene encoding probable ATP-binding/permease fusion ABC transporter), *fepC* (gene encoding ferric enterobactin transport protein), *qnrVC6* (quinolone resistance gene), and two insertion sequences (IS*CR1* and IS*26*). In *bla*_AFM-1_ carrying pHS17-127, the resistance gene cassette array upstream of the IS*CR29*-*bla*_AFM-1_ unit consists of *aacA4*, *bla*_IMP-45_, *bla*_OXA-1_, *catB3*, sul1, *qnrVC6,* and *bla*_PER-1_.

### Effects of AFM-4 on the MICs of β-lactams.

To better understand the novel gene *bla*_AFM-4_ conferring β-lactam resistance, we cloned *bla*_AFM-4_ into the plasmid pGK1900 and then transformed it into E. coli DH5α and P. aeruginosa PAO1. The effects of AFM-4 differed in DH5α and PAO1 (Table 3).

**TABLE 3:** MICs of recombinant strains producing AFM-2 and AFM-4^*a*^

	MICs of antibiotics (mg/L)							
Strains	MEM	IPM	FEP	PIP	CAZ	PTZ	CZA	AZT
DH5α (pGK1900)	≤0.06	0.5	0.5	1	1	1/4	1/4	0.125
DH5α (pGK1900_AFM2)	1	2	8	32	>256	32/4	>256/4	0.06
DH5α (pGK1900_AFM4)	0.5	2	4	16	>256	16/4	>256/4	0.125
PAO1 (pGK1900)	0.5	2	4	8	4	4/4	4/4	4
PAO1 (pGK1900_AFM2)	>128	>128	>256	>256	>256	>256/4	>256/4	4
PAO1 (pGK1900_AFM4)	>128	>128	>256	>256	>256	>256/4	>256/4	4

*^a^*Abbreviations are the same as in Table 2.

In DH5α, AFM-4 increased the MIC levels up to 2–4-fold for meropenem, imipenem, cefepime, piperacillin and piperacillin-tazobactam. The MICs of ceftazidime and ceftazidime-avibactam were elevated over 8-fold by AFM-4. In PAO1, the MICs of all β-lactams increased over 5-fold, except aztreonam, which of course cannot be hydrolyzed by AFM-4, an MBL. We cloned *bla*_AFM-2_ with the same method. The antimicrobial susceptibility testing results showed that there was little difference in the antibiotic resistance effects between AFM-2 and AFM-4.

## DISCUSSION

As a novel MBL, AFM alleles have not been widely disseminated in P. aeruginosa. In multicenter surveillance, only three AFM-producing P. aeruginosa strains were identified from 605 clinical isolates, accounting for 0.50% (8). In our study, AFM-producing strains occupied 1.03% (5/487) of all isolated strains, and four strains isolated from the same patient (out of five AFM-producing strains) had clonal relatedness. AFM-2-producing P. aeruginosa strains belong to ST262 and AFM-4-producing P. aeruginosa AR19438 belongs to ST671, both of which are not common clinical sequence types of CRPA. Compared to the strains without AFM, AFM-producing strains showed higher MICs of β-lactams, fluoroquinolones, and aminoglycosides, as a result of acquiring drug resistant plasmids. To date, ST671 P. aeruginosa has been reported to contaminate rinse water, leading to the bronchoscope-related pseudo-outbreak (12). AR19438 was also isolated from the sink surface, indicating that ST671 P. aeruginosa isolates might be related to the water environment of wards. The contamination of the water environment was closely associated with P. aeruginosa infections in ICUs (13–15). Thus, we should be alerted to the dissemination of the AFM-4-producing ST671 P. aeruginosa clone in the ICU.

In this study, *bla*_AFM-2_ genes were observed to be encoded on plasmids similar to those previously reported in other P. aeruginosa strains (7, 9). However, *bla*_AFM-4_ was located on a pSTY-like megaplasmid, which was different from the IncP-2 plasmids encoding *bla*_AFM1-3_ in P. aeruginosa strains (7, 8). Comparing pAR19438 to pSTY, pAR19438 may have acquired *bla*_AFM-4_ by the integration of the AFM-4-encoding Tn*1403*-like transposon. Only seven pSTY-like megaplasmids were found in the NCBI database, all belonging to Pseudomonas sp. (Table S1), indicating the species specificity of this megaplasmid family. Except for pSTY, other pSTY-like megaplasmids have all been identified in China in recent years. Continuous surveillance of these newly emerged megaplasmids is required due to their ability to be integrated with resistance genes and spread among Pseudomonas sp.

IS*CR*s are linked with variant resistance determinates and are transmitted by rolling circle, which contributes to the rapid dissemination of drug resistance (16, 17). The genetic context of *bla*_AFM_ is an IS*CR29*-*bla*_AFM_ unit core module flanked by class 1 integrons. IS*CR29*-*bla*_AFM_ units are identical in most *bla*_AFM1-3_ carrying plasmids, suggesting that IS*CR29* is responsible for the dissemination of *bla*_AFM_. However, in pAR19438, this core module was interrupted by IS*CR*-*msrB*-*msrA*-*yfcG*-*corA*, which added to its complexity. Although the AFM-producing P. aeruginosa strains were isolated in a single room, the genetic contexts of *bla*_AFM-2_ and *bla*_AFM-4_ varied dramatically, indicating that there was no horizontal transfer of drug resistance genes between strains of the two sequence types.

The simulated structure of AFM was a classic αβ/βα sandwich similar to all MBLs, with Zn1 interacting with histidine residues 117, 119 and 186 and Zn2 interacting with residues Asp121, Cys205 and His247 (7). The mutation sites of AFM-2, AFM-3 and AFM-4 compared to AFM-1 are Pro13 or Ala15, and these sites have no impact on either the overall fold or the active sites (Fig. S2), which could explain the close impact of AFM-2 and AFM-4 on antimicrobial susceptibility.

In conclusion, we identified AFM-producing P. aeruginosa strains in the ICU and analyzed their genetic characteristics. A novel AFM allele, AFM-4, was identified, which was located in the pSTY-like plasmid and embedded in a different genetic context compared to other AFM alleles in P. aeruginosa. The continuous emergence of variant carbapenemase-producing Pseudomonas aeruginosa needs to be properly addressed. And persistent genomic surveillance should be conducted, as carbapenemase genes are widespread.

## MATERIALS AND METHODS

### Isolation and identification of AFM-carrying strains.

P. aeruginosa strains were isolated from environmental and patient samples in an ICU in Zhejiang Provincial People's Hospital in Zhejiang Province, China using Pseudomonas Isolation Agar plates (18). Then we used a Vitek II automatic microbe analysis instrument (bioMérieux) to identify the species. AFM alleles were screened using PCR (PCR) to amplify the full ORF with the primer pairs: AFM-F (5′-cagctctgagattgaggcga-3′) and AFM-R (5′-attgggtgtgacgtggtca-3′). PCR products were sequenced by Sanger sequencing.

### Whole-genome sequencing and analysis of selected strains.

We selected AFM-carrying strains and the strains isolated from the same patient or the same environmental sampling point for whole-genome sequencing. Detailed information on the sequenced strains is listed in Table 1. All eight selected strains were sequenced by the Illumina HiSeq platform. Additionally, three strains (AR19438, AR19583, and AR19640) were selected for nanopore sequencing. The Illumina sequencing raw reads of these strains were assembled by shovill 0.9.0 (https://github.com/tseemann/shovill). Hybrid assembly of Illumina and Nanopore reads was performed by Unicycler v0.4.8 (19). PubMLST (https://pubmlst.org/organisms/pseudomonas-aeruginosa) was used to identify multilocus sequence typing (MLST) of strains. Prokka 1.14.6 was used for gene annotation (20). ABRicate 1.0.0 (https://github.com/tseemann/abricate) was used to identify the resistance genes. BWA-MEM was used to align sequence reads against the plasmid sequence(21). The genetic sequence comparisons were performed and visualized using Easyfig 2.2.5 and BRIG-0.95 (22, 23). The conjugative apparatus was predicted by oriTfinder (https://tool-mml.sjtu.edu.cn/oriTfinder).

### *bla*_AFM_ cloned into P. aeruginosa and E. coli.

*bla*_AFM_ genes and their upstream sequences (a total of 1711 bp) were amplified from AR19438 and AR19640 and cloned into pGK1900 using a Hieff Clone Plus One Step Cloning kit (7). The AFM expressing plasmids were transformed into E. coli DH5α by chemical transformation and into P. aeruginosa PAO1 by electroporation transformation.

### Antimicrobial susceptibility testing.

Antimicrobial susceptibility testing of the isolated strains and cloning experimental strains was performed by the broth microdilution method. The antibiotics used included meropenem (Hanhui Pharmaceutical Co., Ltd., China), imipenem (Merck Sharp & Dohme Corp, USA), cefepime (Jiangsu Hengrui Pharmaceutical Co., Ltd., China), piperacillin (Suzhou Erye Pharmaceutical Co., Ltd. China), ceftazidime (Guangdong Jincheng Jinsu Pharmaceutical Co., Ltd., China), tazobactam (Meilunbio, China), avibactam (MedChemExpress, USA), aztreonam (Sigma-Aldrich, USA), ciprofloxacin (Fluka Analytical, USA), amikacin (Meilunbio, China), gentamicin (Sigma-Aldrich, USA), and colistin (Sigma-Aldrich, USA). The P. aeruginosa strain ATCC27853 was used for quality control. The results were interpreted according to CLSI performance standards (30^th^ Edition) (24).

### Conjugation of plasmids.

Conjugation experiments were performed with AR19438 and AR19640 as donors and a rifampicin-resistant mutant of PAO1 as the recipient (18). The antibiotics and the concentration used for selection were rifampicin (300 μg/mL) and meropenem (8 μg/mL).

### Data statement.

The data in our research are available to access. The genome sequences of P. aeruginosa strains in this study have been deposited in NCBI under the BioProject (Accession: PRJNA826241). The AFM-4 allele has been deposited in NCBI with the accession number NG_079223.
