# Supplementary material for: Plasmid-Borne AFM Alleles in Pseudomonas aeruginosa Clinical Isolates from China
Source: Microbiol Spectr. 2022 Aug 24;10(5):e02035-22. doi: 10.1128/spectrum.02035-22 (PMC9602987; doi:10.1128/spectrum.02035-22)

Table S1 The BLAST results of pAR19438 and other pSTY-like megaplasmids

Description	Max Score	Total Score	Query Cover	E value	Per. ident	Acc. Len	Accession
Pseudomonas sp. CIP-10 plasmid unnamed1	90704	3.50E+05	72%	0	98.81	395258	CP087161.1
Pseudomonas sp. p1(2021b) plasmid unnamed	90699	3.95E+05	76%	0	98.81	337874	CP083747.1
Pseudomonas sp. VLB120 plasmid pSTY	89258	3.69E+05	75%	0	99.98	321653	CP003962.1
Pseudomonas sp. XWY-1 plasmid	36069	3.19E+05	69%	0	99.95	394537	CP026333.1
Pseudomonas monteilii strain QJ20133 plasmid pJ20133-VIM	32465	2.68E+05	61%	0	94.48	255073	MN310371.1
Pseudomonas sp. CFA plasmid unnamed1	29342	2.28E+05	59%	0	92.92	201121	CP044547.1

Figure S1. The Amino acid sequence alignment of AFM-1, AFM-2, AFM-3 and AFM-4

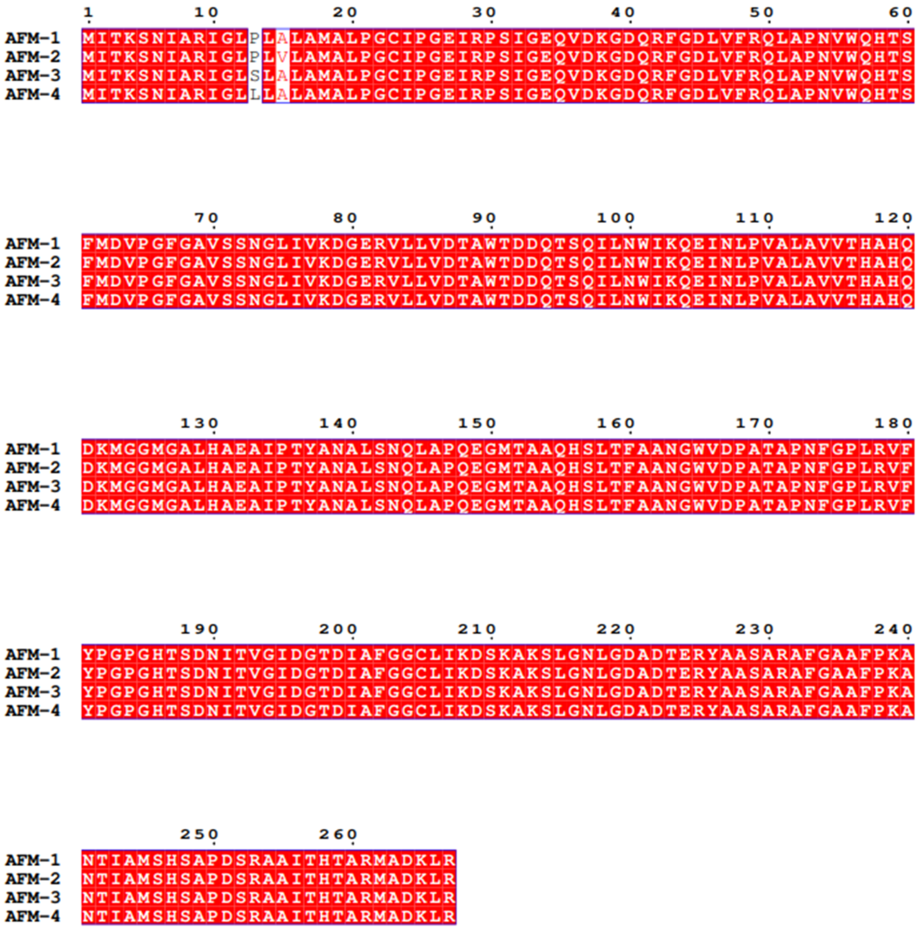

Figure S2. The overall modelling structure of AFM alelles

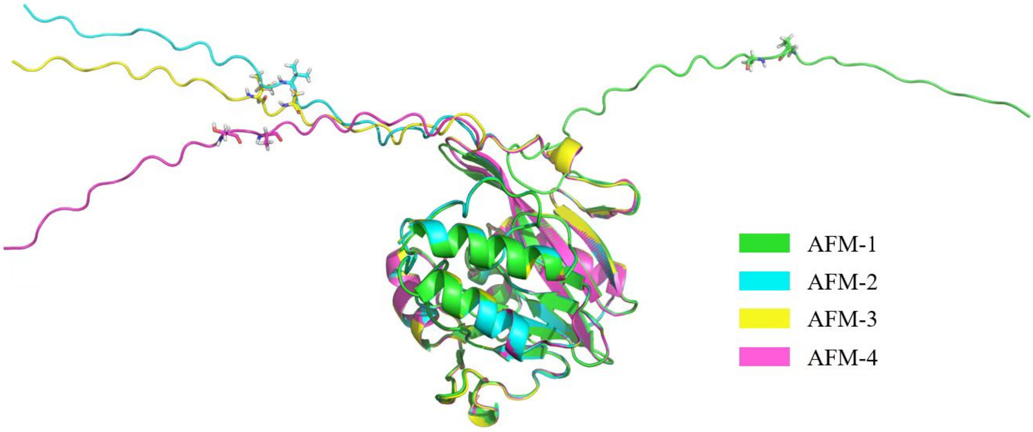

Supplement: Supplemental file 1 — Supplemental material. Download spectrum.02035-22-s0001.pdf, PDF file, 1.5 MB [file spectrum.02035-22-s0001.pdf]
